# Population Size Estimation Methods: Searching for the Holy Grail

**DOI:** 10.2196/25076

**Published:** 2020-12-03

**Authors:** Joyce J Neal, Dimitri Prybylski, Travis Sanchez, Wolfgang Hladik

**Affiliations:** 1 Epidemiology and Surveillance Branch, Division of Global HIV & TB Center for Global Health Centers for Disease Control and Prevention Atlanta, GA United States; 2 Rollins School of Public Health Emory University Atlanta, GA United States

**Keywords:** HIV, key populations, population size estimation, capture-recapture

## Abstract

Accurate size estimates of key populations (eg, sex workers, people who inject drugs, transgender people, and men who have sex with men) can help to ensure adequate availability of services to prevent or treat HIV infection; inform HIV response planning, target setting, and resource allocation; and provide data for monitoring and evaluating program outcomes and impact. A gold standard method for population size estimation does not exist, but quality of estimates could be improved by using empirical methods, multiple data sources, and sound statistical concepts. To highlight such methods, a special collection of papers in JMIR Public Health and Surveillance has been released under the title “Key Population Size Estimations.” We provide a summary of these papers to highlight advances in the use of empirical methods and call attention to persistent gaps in information.

Globally, most new HIV infections in 2019 were estimated to have occurred among key populations (KP), including sex workers, people who inject drugs (PWID), transgender people, and men who have sex with men (MSM), as well as their partners [1]. Worldwide, 62% of new HIV infections among adults were attributed to KP and their partners, ranging from 28% of new infections in eastern and southern Africa to 99% in Eastern Europe and central Asia [1]. Inferences such as these require not only robust analyses for the number of people living with HIV but also accurate size estimates for the various at-risk populations. For decades, population size estimation (PSE) has suffered from the lack of a gold standard method, leading to the use of numerous techniques and approaches with varying robustness and implemented with erratic fidelity [2]. Underestimates are believed to be common, but because we do not have actual population size counts, they are typically accepted and used for the sake of political expediency.

Accurate estimates of KP size are essential for understanding the scale of the response required to ensure adequate availability of services needed to prevent or treat HIV infection; to inform HIV response planning, target setting, and resource allocation; and to provide data for monitoring and evaluating program outcomes and impact. For example, KP size estimates could help measure progress toward the Joint United Nations Programme on HIV/AIDS (UNAIDS) 95-95-95 goals (95% of HIV-positive individuals know their status; of these, 95% are receiving antiretroviral therapy; and of these, 95% are virally suppressed) [3]. However, the availability and quality of KP size estimates vary globally. Many countries have conducted PSE exercises, but results often are buried in surveillance reports (ie, these estimates may not be published in journals or presented at conferences) [4]. UNAIDS and the Centers for Disease Control and Prevention (CDC) have worked to compile data from PSE conducted as standalone studies or as part of biobehavioral surveys (BBS) [2,5,6]. Often, however, reported PSE are based on methods that are neither empirical (based on scientific, systematic observation or measurement) nor standardized and are not well documented. Furthermore, KP size is frequently reported as a point estimate without specifying measurements of statistical variability, such as confidence limits or credible intervals. Although estimates derived from nonempirical methods (ie, based on opinion or nonsystematic observation) such as the Delphi method, wisdom of the crowds, and hotspot mapping may be useful for programmatic planning, more robust empirical PSE methods generally can be expected to facilitate better estimates of the number of KP members living with HIV (KPLHIV), yielding more representative and higher quality data [7,8] for use in measuring progress toward various targets, including percent of KPLHIV aware of their HIV status, percent of KPLHIV receiving antiretroviral treatment, and percent of those virally suppressed. The general population is relatively easy to enumerate using census methods, but estimating the size of KP faces several challenges: lack of a sampling frame, mobility or economic migration, and some KP members may not want to be counted (ie, they may choose to be less visible because of the stigma or criminalization of their KP-defining behaviors) [9,10]. In the absence of a gold-standard PSE method, methods that use empirical data, multiple data sources, and sound statistical concepts can be expected to provide more valid estimates than nonempirical methods.

Focused on application of empirical methods, a special collection of papers in *JMIR Public Health and Surveillance* has been released under the title “Key Population Size Estimations” [11]. These 9 reports on empirically based PSE include innovative approaches, such as use of social media apps (Vietnam) [12], a reverse-tracking method (Namibia) [13], multiple-source capture-recapture (CRC; Uganda) [14], and successive sampling (SS)-PSE incorporating imputed visibility [15]. We provide a summary of these papers to highlight advances in the application of empirical methods and persisting gaps in information.

Almost all the papers (Table 1) presented estimates based on some form of CRC methodology — conventional two-source (2S)-CRC; multiple-source CRC; or service, unique object, unique event, or social app multiplier methods — or used prior estimates that may have been partially based on these methods for analysis (SS-PSE). Of the 9 papers, 4 described PSE embedded within a BBS [13,16-18], 1 described using datasets from previously conducted BBS [15], and 4 presented results from standalone PSE exercises [12,14,19,20].

**Table 1:** Population size estimation methods, key populations, and geographic location covered by papers in the special-themed issue.

Methods	Key population	Location	Citation
2S-CRC^a^	FSW^b^, MSM^c^	Uganda, 11 towns	19
2S-CRC	FSW (venue-based)	Vietnam, Ho Chi Minh City	20
Social app multiplier method	MSM	Vietnam, 12 provinces	12
3S-CRC^d^	FSW, MSM, PWID^e^	Uganda, Kampala	14
3S-CRC; multiplier method	FSW	South Sudan, 2 cities	16
Multiplier methods	FSW	South Africa, 3 cities	17
Multiplier methods, SS-PSE^f^	FSW, MSM/TGW^g^	Papua New Guinea, 3 cities	18
SS-PSE	FSW, MSM, PWID	Armenia, 3 cities	15
RTM^h^, RadR^i^	FSW	Namibia, 2 cities	13

^a^2S-CRC: 2-source capture-recapture.

^b^FSW: female sex workers.

^c^MSM: men who have sex with men.

^d^3S-CRC: 3-source capture-recapture.

^e^PWID: people who inject drugs.

^f^SS-PSE: successive sampling population size estimation.

^g^TGW: transgender women.

^h^RTM: reverse tracking method.

^i^RadR: respondent-driven sampling–adjusted RTM.

Apodaca and colleagues [19] conducted a 2S-CRC in 11 small Ugandan towns with peers distributing unique objects to tag female sex workers (FSW) and MSM in the first capture. Distributers used a mobile global positioning system to record locations of the distribution for quality control purposes. A different group of peers (to minimize the risk of visiting the same venues again) collected data for the second capture, which consisted of asking FSW and MSM if they had received the unique object using 2 different recapture definitions: presentation of the object or identification of the object from a set of photos. The most credible results (compared with other published estimates) were based on presentation of the object. Among the first empirically based PSE to be done in Uganda to obtain FSW and MSM size estimates at the small-town level, this exercise demonstrated the difference in results based on recapture definitions and the feasibility of using peers for data collection when provided proper training and standardized data collection tools.

To estimate the size of venue-based FSW in Ho Chi Minh City, Vietnam, Le et al [20] conducted multistage 2S-CRC. They used stratified probability proportionate to size to select districts, mapped venues, and distributed unique objects to all FSW in those venues. The recapture consisted of an equal probability random selection of venues from the initial mapping and asking FSW in those venues if they had received the object. The PSE of venue-based FSW in these districts was multiplied by the inverse of the proportion of districts selected to calculate the number of venue-based FSW in Ho Chi Minh City. Although this PSE method is useful for venue-based KP, the authors note that estimates are needed for other FSW, including those who may seek clients using social media platforms.

Son et al [12] used a social media app multiplier method for PSE of MSM in 12 provinces in Vietnam. The first data source was the count of social app users, and the second source was data collected from MSM recruited via respondent-driven sampling (RDS) and who responded to an online questionnaire (telephone survey for MSM who did not have internet access). The PSE was derived by dividing the number of app users in a 1-month period by the proportion interviewed who reported using the app during the same period. Investigators estimated the size of the MSM population in 12 provinces, from which they extrapolated to generate a national PSE among MSM aged 15-49 years. This first attempt to estimate the MSM population in Vietnam empirically highlighted the feasibility of reaching many MSM through a social app and online. The percentage of men estimated to be MSM nationally (0.68%, 95% CI 0.46%-1.95%) is well below the published estimates for Southeast Asia of 3%-6% of MSM in the past 12 months [21]. PSE may have been underestimated by selecting users of only 1 social app, being biased toward those with higher internet or social app literacy, excluding MSM aged ≥50 years, and assuming that users of the social app during a 1-month period represented all MSM. Reported PSE may represent a minimum or a subgroup of MSM — some provinces had population proportions that were improbably low (eg, 0.21%). Future efforts should try to achieve better precision — wide confidence intervals included crude estimates previously derived by nonempirically based consensus methods. In one province, the PSE confidence interval ranged from about 4200 to 68,000, representing 0.6% to 9.6% of the male population aged 15-49 years. Adapting traditional empirical methods using social apps and web-based interviews, this method is quick and relatively inexpensive but needs improvement and additional validation.

In a study (published elsewhere) on the uptake of KP PSE in guiding HIV responses in Africa, Viswasam et al [4] described limited uptake of PSE in US President’s Emergency Plan for AIDS Relief (PEPFAR) Country Operational Plans, national strategic health planning documents, and Global Fund Concept Notes and recommended stakeholder engagement and data-oriented capacity building. Two papers in this special-themed issue described implementation of multiplier methods in conjunction with BBS and the importance of stakeholder engagement in 3 South African cities among FSW [17] and in 3 Papua New Guinea cities among MSM, transgender women (TGW), and FSW [18]. Investigators in South Africa used unique object, unique event, and service multiplier methods; wisdom of the crowd; and a modified Delphi method to adopt consensus estimates [17]. Asserting that a PSE has limited value unless it is adopted and used by government, civil society, and global health funding partners, Grasso et al [17] found that stakeholder engagement and consensus were critical to vetting and triangulating multiple empirically based estimates to ensure adoption and use of the PSE at the national and subnational levels. Because it is equally important not to allow political expediency or agendas to adjust what otherwise would be the best available PSE, data-oriented capacity building as promoted by Viswasam et al [4] may be essential to prevent the adoption of inferior PSE.

The Papua New Guinea PSE exercise, part of a BBS conducted among MSM and TGW (combined) and FSW, employed unique object and service multiplier methods as well as SS-PSE [18]. As in South Africa, final estimates were chosen through meetings among experts that were then presented to key stakeholders for adoption of a single estimate in each city. Authors highlighted the challenges in using these methods — the wide variation in results and importance in understanding the biases in data collection, including issues with the availability and quality of HIV-service data.

Doshi et al [14] demonstrated the feasibility of using 3-source (3S)-CRC as a standalone (ie, not done in conjunction with a survey) method for PSE in a resource-limited setting [14]. One of the benefits of 3S-CRC is the ability to account partially for sample dependencies (thereby relaxing the assumption of independence required by 2S-CRC methods) by allowing sources to be examined pairwise. In this first use of 3S-CRC for FSW, MSM, and PWID in Kampala, Uganda, the project team distributed 2 different unique objects in each of the first 2 captures, C1 and C2. KP members were asked in C2 and C3 whether they received the objects distributed in C1 and C2. The number in C3 receiving one or both objects was determined. Among PWID, recording errors prevented use of data collected in C3; however, data from C1 and C2 could be analyzed as conventional 2S-CRC. PSE were derived using the Lincoln-Petersen method for 2S-CRC (PWID) and a Bayesian nonparametric latent-class model for 3S-CRC (MSM and FSW). For the latter, statistical analyses were performed in R using the Bayesian nonparametric latent-class capture-recapture package (LCMCR). Use of LCMCR was innovative because this approach was not originally developed for analyzing epidemiologic data.

Okiria et al [16] also described exercises undertaken in conjunction with a BBS to estimate the number of FSW in the South Sudan capital city, Juba, and Nimule, on the South Sudan-Uganda border. They used unique object and service multiplier methods as well as 3S-CRC. The attempt to conduct 3S-CRC in Juba was thwarted when the third capture, delayed because of unique object procurement issues, was conducted 6 months after the BBS data collection had concluded and did not include questions needed to determine the number of individuals in each capture separately. Therefore, analysis was treated the same as for a routine unique object multiplier method. In Nimule, they found divergent PSE across all methods and postulated violation of the closed population assumption because of displacement of FSW due to conflict that delayed BBS launch for 6 months. Furthermore, some results generated implausible FSW population proportions (ie, as high as 193%), possibly due to many FSW not actually residing in Nimule but across the border in Uganda. Lessons learned included the need to improve data quality during collection (eg, ensuring correct identification of residency and deduplication of service records) and timing the first 2 captures before the BBS (if BBS is used as a data source) to ensure individual-level data can be more accurately collected by interviewers who receive intensive training compared with volunteer object distributors. Despite conflict and logistical and operational challenges, investigators demonstrated the feasibility of conducting 3S-CRC and found that use of multiple methods to estimate the number of people not easily counted during mapping improved PSE compared with previous results.

To advance the SS-PSE method [22], McLaughlin et al [15] examined the performance of a modification that allows visibility to be jointly modeled with population size. Imputed visibility is a measure of how likely persons are to participate in an RDS survey [23]. This measure may be used instead of self-reported social network size, which is usually considered a proxy for inclusion probability. Using 15 datasets from RDS surveys of FSW, MSM, and PWID from 3 cities in Armenia, they compared and evaluated the accuracy of imputed visibility PSE against those found for the same populations based on other methods. The imputed visibility adjustment worked well with great (as defined by authors) fits with prior estimation for FSW and PWID, but MSM populations in all 3 cities had inconsistencies with expert prior values that made a great fit impossible. Authors cautioned that prior estimations from expert opinions may not always be accurate and that SS-PSE be used only after ensuring that RDS assumptions have been met, convergence has been reached on primary endpoints, and the sampled population network structure does not have bottlenecks. Lastly, to ensure generation of the most accurate estimates, they recommended that SS-PSE be used in conjunction with other PSE commonly used in RDS surveys as well as with other years of SS-PSE.

Wesson et al [13] described using an RDS adjustment (respondent-driven sampling–adjusted [RadR]) to the less-commonly used reverse tracking method (RTM) [24] to estimate the population size of FSW in Namibia [13]. The RadR method allows for application in RDS surveys and improves upon venue-based RTM because RaDR-based results account for the proportion of KP that do not congregate at venues and thus should provide more representative PSE. Additionally, RadR can adjust for double-counting associated with traditional venue-based RTM. The novel RadR method was successfully integrated with RDS surveys among FSW in 2 sites in Windhoek, Namibia to provide plausible PSE estimates compatible with the Namibia Ministry of Health and Social Services official estimates based on triangulation of PSE derived from multiple methods. This study demonstrated that, although it still needs more field testing with other KP groups and in other geographical settings, the RadR method is easy to integrate into RDS surveys and might be a promising methodological advance.

Of the 9 papers in this collection, 8 papers described PSE of FSW, 2 reported on PWID, and 5 included MSM (of these, 1 combined TGW with MSM). TGW PSE are lacking globally [2,4-6,8]. Viswasam et al [4] conducted a review of all PSE for KP in 54 African countries from 2009 through 2017 and identified 118 size estimates — 70 for FSW, 27 for MSM, 21 for PWID, and none for transgender persons. PSE frequently combines TGW with MSM; because the 2 populations have distinct needs, generating separate estimates will be crucial for advocating for both KPs and appropriately targeted programs and services. Although only 2 papers in this collection reported PWID size estimates [14,15], many countries have included or are planning to include PSE in their BBS among PWID [25,26; personal communication]. Estimating the size of the PWID population may be less challenging in areas with substantial drug-injecting activity, for example, along drug transportation routes and border areas. A dearth of size estimates for the female PWID population argues for enhancing efforts to recruit female PWID for BBS and PSE. Challenges to estimating population size of FSW include their mobility, driven by economic needs and opportunity, and the variety of settings in which they work or find clients, including the increasing use of internet sites and social media apps. Social media and dating apps are emerging as a data source for PSE, especially among MSM [12,27], but should be used with caution. Limitations of social media and dating apps for size estimation include potential overcounting if many users are allies or onlookers and not members of the KP and if identification and deduplication of users with multiple accounts are not possible; dependence on internet and smart phone coverage; variability of app use by age and cultural context; and unstable popularity of apps as new apps are introduced and adopted. Furthermore, detailed information about how social apps derive estimated number of users and classify user characteristics may be unknown or undisclosed to protect proprietary interests.

Publications of KP PSE report the estimated absolute number of KP but frequently omit the KP population proportion. This metric, however, provides a reality check to assess underestimation or overestimation by comparison with other published estimates [5,6,8,21,28]. For example, 1.5% may be a reasonable minimum threshold for an urban MSM population proportion — figures less than 1.5% suggest underestimation. The proportion of the population who are KP members will vary by characteristics (eg, urbanicity, border, transportation routes, drug trafficking routes, economic opportunity). The proportion of the population that is MSM or TGW probably is the most stable compared with that for PWID or FSW, which is more variable and localized. The principal challenge, however, is to account for MSM mobility (ie, migration from rural to urban areas and to an unknown extent, small-to-larger town migration). Hence, the proportion of MSM among urban men can and should be expected to be higher as they absorb MSM from rural and small-town settings.

Countries are encouraged to publish PSE reports in the peer-reviewed literature, in addition to including estimates in their surveillance reports or reporting estimates to UNAIDS and other agencies upon request. Even when published, however, Viswasam and colleagues [4] noted that there remains “limited evidence of sustained uptake of these data to guide the HIV responses.” In another review of the available and quality of KP size estimates, Sabin et al [2] concluded that size estimates are “increasingly available but quality varies widely” and that “different approaches present challenges for data use.” This collection of papers provides examples of PSE reports that may serve as models for countries to use to publish their own results. We recommend that countries use multiple, robust empirical methods; document the process; synthesize results; report point estimates with confidence or credible intervals; include population proportions (using appropriate sex-specific, age-specific, and location-specific census data); and take steps to ensure uptake and use of estimates to guide the HIV response toward ending the HIV epidemic among KP.

Numerous challenges remain, including the aforementioned need for distinct PSE for TGW and female PWID. If a BBS is deemed impractical because of relatively small population sizes or lack of resources, standalone PSE exercises as described in 4 of the papers [12,14,19,20] presented in this special-themed issue (described earlier) may be considered to fill these gaps. We continue to seek new, innovative, or improved methods in a search for the holy grail (ie, a gold standard for finding the true population size). In addition to encouraging publication and use of high-quality PSE, we underscore the need for a global consensus on minimum-threshold PSE to prevent the use of extreme underestimates and highlight the continued need for operational research to advance empirically based PSE.

## Abbreviations

2S-CRC: 2-source capture-recapture
3S-CRC: 3-source capture-recapture
BBS: biobehavioral surveys
CDC: Centers for Disease Control and Prevention
FSW: female sex workers
KP: key populations
KPLHIV: key populations living with HIV
LCMCR: latent-class capture-recapture package
MSM: men who have sex with men
PEPFAR: President’s Emergency Plan for AIDS Relief
PSE: population size estimation
PWID: people who inject drugs
RadR: respondent-driven sampling–adjusted reverse tracking method
RDS: respondent-driven sampling
RTM: reverse tracking method
SS-PSE: successive sampling population size estimation
TGW: transgender women
UNAIDS: Joint United Nations Programme on HIV/AIDS
